# High-Temperature Sensitivity in Stimulated Brillouin Scattering of 1060 nm Single-Mode Fibers

**DOI:** 10.3390/s19214731

**Published:** 2019-10-31

**Authors:** Sanggwon Song, Aeri Jung, Kyunghwan Oh

**Affiliations:** Institute of Physics and Applied Physics, Yonsei University, Seoul 120-749, Korea; sgsong@yonsei.ac.kr (S.S.); aerij37@yonsei.ac.kr (A.J.)

**Keywords:** single mode optical fiber, temperature measurement, Brillouin scattering

## Abstract

With the rapid advancement of Yb-doped fiber lasers (YDFL) whose output wavelength is near 1060 nm, passive fibers to carry the high optical power at the spectral range are also gaining significant importance. Stimulated Brillouin scattering (SBS) in the passive fibers connecting components in the lasers, especially, can set a fundamental limit in the power handling of YDFL systems. We experimentally analyzed SBS characteristics of passive single mode fibers (SMF) at a wavelength of 1060 nm. For two types of SMFs (Corning HI1060 and HI1060Flex), the Brillouin frequency (*ν_Β_*), its linewidth (Δ*ν_Β_*), and their variations with respect to the input laser power and the surrounding temperature were experimentally measured, along with the SBS threshold power (*P_th_*). The optical heterodyne detection method was used to identify temperature-dependent SBS characteristics of fibers, and we found SMFs at λ = 1060 nm showed a temperature sensitivity in SBS frequency shift more than 40% higher than in conventional SMFs operating in C-band. Detailed procedures to measure the SBS properties are explained, and a new potential of 1060 nm SMF as a distributed temperature sensor is also discussed.

## 1. Introduction

The stimulated Brillouin scattering (SBS) has been attributed as one of the most fundamental limiting factors in the power scaling of rare earth-doped fiber laser systems [1,2,3,4]. The SBS converts the forward-propagating laser power into the backward-propagating and frequency-shifted SBS light over the whole fiber propagation length by a nonlinear optic process, which not only limits the laser transmitting power, but also can jeopardize all the preceding optical components’ functionalities. In recent years, Yb-doped fiber lasers (YDFLs) lasing near λ = 1060 nm have been extensively studied to achieve an optical power over the tens of kilowatt level in the continuous wave (CW) operation, which could find various applications in material processing, directional energy weapon systems, as well as in biomedical therapeutics [5,6,7]. The SBS within YDFL cavities has been intensively investigated and various geometrical [8] and material structures [9] of active fibers have been proposed to suppress the SBS, along with external modulation of the incident light in a certain modulation format [10].

Yb-doped fiber laser/amplifier output should be carried through “un-doped passive” fibers and there have been various types of beam delivery fiber and cables for high power applications [11]. Besides this high power beam delivery, there exists an ever-increasing need for a single-mode passive fiber near λ = 1060 nm that can carry light with a moderate power for optical component connection, laser monitoring, and optical feedback control to maintain the stability in the optical power and the spectral position of high power YDFLs [12,13]. In order to cope with these demands, passive single-mode fibers (SMFs) at λ = 1060 nm have been developed and are commercially available. Even though the power along these 1060 nm SMFs is relatively lower than in YDFLs and amplifiers, the power handling level within these fibers continues to increase reaching well above the SBS threshold. Despite these growing concerns, detailed experimental analysis of the SBS along the passive 1060 nm SMFs has not been reported. In contrast, the SBS parameters have been well quantified in various SMFs operating in the C-band [14,15,16,17,18] for telecommunications and for distributed optical sensing applications [19,20,21,22]. Especially, distributed temperature sensing over conventional SMFs has found various practical applications to monitor the temperature profiles over a long length of installed optical fiber cables [21,22,23,24,25]. With a rapid light source development near λ = 1060 nm in recent years [26,27,28], and matching 1060 nm SMFs on the market [29,30], it is a good time to investigate the SBS characteristics of 1060 nm SMFs and their variation with respect to temperature in order to find potential for enhancing their distributed temperature sensing capability.

To the best of our knowledge, we report for the first time systematic experimental characterizations of the SBS in passive 1060 nm SMFs using a highly sensitive optical heterodyne method with a narrow linewidth CW laser operating at λ = 1064. For two types of commercially available standard passive 1060 nm SMFs (Corning HI1060, and HI1060Flex), we experimentally investigated the Brillouin frequency (*ν_Β_*), the linewidth (Δ*ν_Β_*), the relative peak power, the SBS threshold powers (*P_th_*), and their variations with respect to the input laser power. We report the thermal variations in SBS characteristics of 1060 nm SMFs for the first time and we further discuss the potential of distributed temperature sensing along 1060 nm SMFs with an enhanced sensitivity compared with conventional SMFs in C-band.

## 2. Physical Principles and Experiments

The Brillouin frequency *ν_Β_* depends on both the optical and the acoustic properties of the optical fiber and is given as [14]

(1) $$v_{B}=\frac{2n_{eff}V_{a}}{\lambda_{L}},$$

where *n_eff_* is the effective index of the guided mode, *λ_L_* is the incident laser wavelength, and *V_a_* is the fiber’s acoustic velocity. The Brillouin gain coefficient *g_B_* is expressed as [31]

(2) $$g_{B}=g_{B0}\frac{{\left(\Delta v_{B}\right)}^{2}}{{\left(v-v_{B}\right)}^{2}+{\left(\Delta v_{B}\right)}^{2}},\;g_{B0}=\frac{2\pi \gamma^{2}}{nc\rho_{0}V_{a}\lambda_{L}^{2}\Delta v_{B}K},$$

where *γ* is the electrostriction coefficient of silica glass, *c* is the speed of light in a vacuum, *ρ_0_* is mean mass density of silica, *K* is the polarization factor, and Δ*ν_Β_* is the full-width at half maximum(FWHM) linewidth of the SBS spectrum. The SBS threshold power *P_th_* is estimated as [32]

(3) $$P_{th}=21\frac{A_{eff}}{L_{eff}g_{B}}\left(1+\frac{\Delta v_{L}}{\Delta v_{B}}\right),$$

where *A_eff_* is the effective modal area, *L_eff_* is the effective interaction length, and Δ*ν_L_* is the incident laser linewidth. As shown in the Equation (3), the SBS threshold depends on *L_eff_*. The SBS threshold could be further lowered if we increase the fiber length but the temperature sensitivity would not be significantly affected.

**Figure 1:**
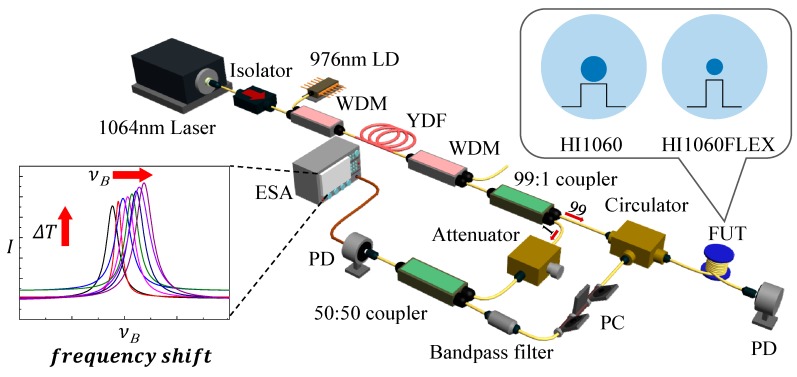
Schematic diagram of the all-fiber optical heterodyne SBS measurement set-up for passive 1060 nm single mode fibers (SMFs) (Corning HI1060, HI1060Flex) (YDF: Yb-doped fiber; WDM: wavelength division multiplexer; LD: laser diode; PD: photodetector; PC: polarization controller; FUT: fiber under test; ESA: electric spectrum analyzer).

In this study, these SBS parameters were experimentally quantified for two 1060 nm SMFs, Corning HI1060, and HI1060Flex using a highly sensitive optical heterodyne technique [33]. The experimental set-up is schematically presented in Figure 1. Equal lengths of 500 m were used for HI1060 and HI1060FLEX as a fiber under test (FUT) to analyze and compare the SBS characteristics of the two fibers. A stable narrow linewidth CW laser (CrystaLaser, CL1064-100-S) was used as a reference light at λ = 1064 nm with the laser linewidth of <2.65 MHz. The laser was further amplified in a forward-pumped Yb-doped fiber amplifier (YDFA) using a pump laser diode (LD) at λ = 976 nm. The pump was launched to YDF using a wavelength division multiplexer (WDM). At the YDFA output, another WDM was used to filter out the remaining 976 nm pump. The output of the YDFA was further split using a 99:1 coupler. The 99% port signal passed through a circulator and was launched to FUT generating the backward-propagating SBS signal. The SBS signal whose frequency was shifted by *ν_Β_* was then passed through a narrow bandpass filter to remove the unwanted spectral noise. The bandpass filter had a spectral width of 2 nm centered at λ = 1064 nm. A polarization controller (PC) was used to optimize the polarization states of the SBS signal, which was then combined with the 1% port signal in a 50:50 coupler to create beating between them.

## 3. Experimental Results and Discussion

**Figure 2:**
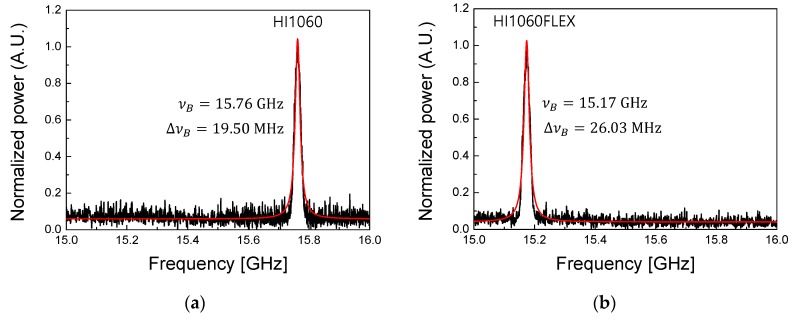
Stimulated Brillouin scattering (SBS) spectra measured by the experimental setup in Figure 1. The two 1060 nm SMFs, (**a**) HI1060 and (**b**) HI1060FLEX, had the equal length of 500 m and the measurements were made at the room temperature, the red curves are the Lorentzian fitting. Here the signal power incident to FUT was about 55 mW at λ = 1064 nm.

**Figure 3:**
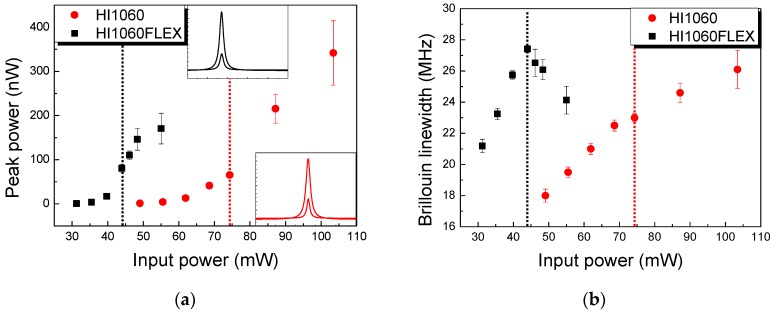
Variation of (**a**) SBS peak power and (**b**) linewidth Δ*ν_Β_* as a function of the input laser power at λ = 1064nm in the room temperature for HI1060 and HI1060FLEX fiber with the length of 500 m.

The beating between the frequency-shifted SBS and the original laser was measured by a fast photodetector (PD) in an electrical spectrum analyzer (ESA), which is a standard heterodyne detection scheme [33]. The results are summarized in Figure 2 for two fibers. Both spectra fit well to the Lorentzian function as in Equation (2) and we obtained *ν_Β_* = 15.76 and 15.17 GHz for HI1060 and HI1060FLEX, respectively. The difference in *ν_Β_* of these fibers was experimentally distinguishable and was attributed to the fiber waveguide structures as provided in the fiber datasheets [29] and the corresponding material compositions. According to the specification, the nominal core-cladding refractive index differences (Δ) were 0.48 and 1.0% for HI1060 and HI1060FLEX, respectively, while the core diameters were 5.3 and 3.4 μm. The mode field diameter was 6.2 and 4.2 μm in HI1060 and HI1060FLEX, respectively. It is noted that HI1060FLEX has a smaller core diameter and a higher Ge concentration than those of HI1060 [34]. These parameters would affect both the acoustic velocity and the effective index to result in a difference in *ν_Β_* (see Equation (1)). At the input power of 55 mW at λ = 1064 nm, the spectral widths Δ*ν_Β_* were measured to be 19.50 MHz and 26.03 MHz for HI1060 and HI1060Flex, respectively. The results were in a good agreement with the prior reports on SMFs in the C-band showing that *ν_Β_* decreased while Δ*ν_Β_* increased as the Ge concentration increased [35]. According to a prior report on C-band SMFs [36,37,38,39], Δ*ν_Β_* changed with the input pump laser power, the waveguide structures, and the Ge doping concentration. We measured the variation of Δ*ν_Β_* as a function of the input pump laser power at λ=1064 nm in the power range of 30 to 105 mW at room temperature, and the results are summarized in Figure 3.

The SBS peak power increased monotonically with an increasing input laser power at λ = 1064 nm in both fibers as shown in Figure 3a. Note that in both cases, *ν_Β_* did not change within the experimental error as indicated in the inset graphs of Figure 3a. The linewidth Δ*ν_Β_* showed a linear monotonic increase in HI1060, whereas in HI1060Flex Δ*ν_Β_* showed a maximum and decreased as the input laser power increased as in Figure 3b. We noted the correlation between the peak SBS power and Δ*ν_Β_* is notably high such that Δ*ν_Β_* significantly changed as in Figure 3b when the slope of SBS peak changed in Figure 3a. See the dotted vertical lines in Figure 3a–b. Detailed variations in SBS linewidth in the 1060 nm SMFs were quite different from those of SMFs in the O,C-band [14,36] which were attributed to the difference in the waveguide structure and material composition.

**Figure 4:**
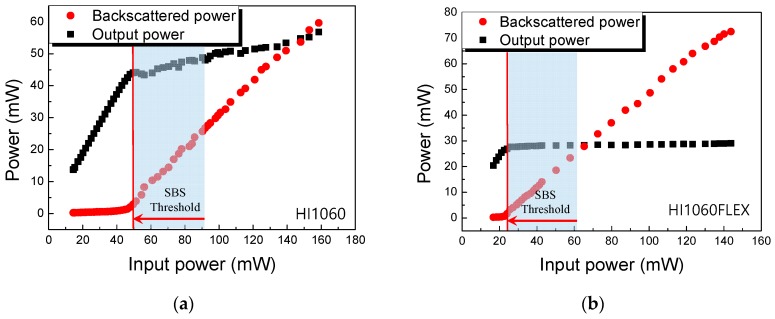
SBS threshold power measurements for (**a**) HI1060 fiber and (**b**) HI1060FLEX fiber.

The threshold of SBS, *P_th_*, was further measured by monitoring the backscattered optical power. The results are summarized in Figure 4. Beyond the threshold, the backscattered power linearly increased while the transmitted output power started to saturate. *P_th_* of 50 and 24 mW was observed for HI1060 and HI1060FLEX, respectively. As expected from the fiber specifications, the *P_th_* of HI1060FLEX was significantly lower than that of HI1060 due to the smaller effective modal area in HI1060FLEX.

It is well known that the Brillouin frequency,*ν_Β_*, of C-band SMFs shifts as a function of both the temperature and the strain, which has been widely applied in distributed sensing technologies over installed fibers and fiber cables [39,40,41]. *ν_Β_* in conventional SMFs in O/C bands has been known to show a linear dependence on the temperature, which is related with the variation in the effective refractive index, the material density, and Young’s modulus [42,43] of silica fiber with respect to temperature. 

**Figure 5:**
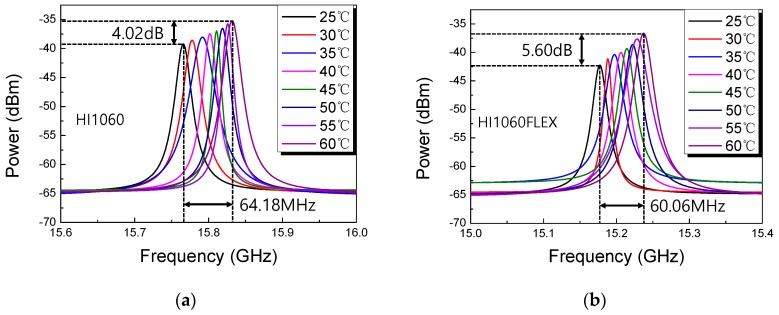
Measurements of Brillouin spectra as a function of temperature. (**a**) HI1060, (**b**) HI1060FLEX. Here the input signal power was 55 mW at λ = 1064nm and the fiber lengths were 500m.

The 1060 nm SMF spools were placed in a temperature-controlled chamber and their SBS spectra were measured at temperatures ranging from 25 to 60 °C. The results are summarized in Figure 5. Here the input laser power was fixed at 55 mW and no external strain was applied. The Brillouin frequency *ν_Β_* increased monotonically with the temperature such that the total *ν_Β_* shift of HI1060 fiber was 64.18 MHz, and that of HI1060FLEX fiber was 60.06 MHz within the observed temperature range. It was also observed that the peak power of the SBS spectra increased monotonically with temperature with the total increase of 4.02 dB and 5.60 dB for HI1060 and HI1060FLEX, respectively. However, the changes in Δ*ν_Β_* did not show a good correlation with the temperature similar to prior reports in conventional SMFs [41,44].

**Figure 6:**
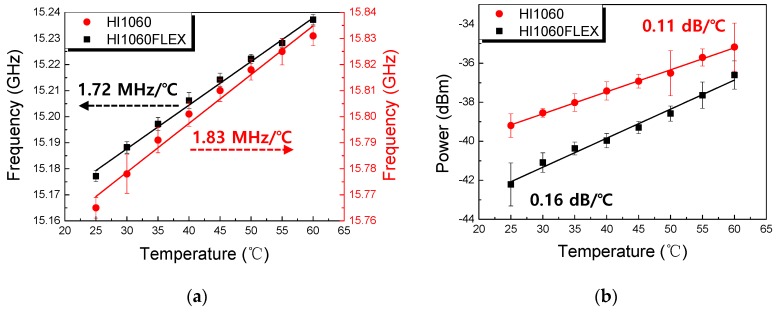
Temperature-dependent Brillouin peak parameters for HI1060 and HI1060FLEX fibers with the incident power of 55 mW. (**a**) The Brillouin frequency *ν_Β_* and (**b**) the peak power. The fiber lengths were 500 m.

In Figure 6 the temperature-dependent Brillouin peak characteristics of 1060 nm SMFs are summarized in terms of *ν_Β_* and its peak power, which showed a good linear response with respect to temperature. In Figure 6a, the temperature-dependent Brillouin frequency shift (*dν_Β_/dT*) of 1.83 MHz/°C and 1.72 MHz/°C were obtained in HI1060 and HI1060FLEX, respectively. The corresponding peak power changes (*dI_peak_/dT*) were 0.11 dB/°C and 0.16 dB/°C.

We compared the temperature-dependent SBS characteristics of conventional SMFs with those of our measurements in Table 1. Conventional fibers in C-band showed a temperature sensitivity, *dν_Β_/dT*, in the range of 0.99 to 1.35 MHz/°C. In the case of temperature annealed C-band SMF *dν_Β_/dT* showed the minimum value of 0.42 MHz/°C [45]. We found that *dν_Β_/dT* of the 1060 nm SMFs (HI1060, HI1060FLEX) in this study was significantly larger than those of C-band SMFs [36,41,45,46,47,48] operating near λ = 1550 nm by more than 40%, which is a notable and meaningful increase in the temperature sensitivity. In the case of O-band operation near λ = 1320 nm, conventional SMF showed *dν_Β_/dT*~1.36 MHz/°C [43] slightly larger than those of C-band SMFs.

As presented in Equation (1), the Brillouin frequency shift is affected by the incident laser wavelength *λ_L_*, effective index of the guided mode *n_eff_*, and acoustic velocity *V_a_*. The temperature sensitivity, therefore, would scale with the inverse of *λ_L_* and would be also affected by the temperature dependence of *n_eff_* and *V_a_*. Detailed attributes of the higher temperature sensitivity of 1060 nm SMFs are being investigated by analyzing the waveguide structure and material compositions to estimate *n_eff_* and *V_a_* by the authors.

In addition, the waveguide structure, Ge concentration in the core, and fiber drawing tension of HI1060 and HI1060FLEX could contribute to the large *dν_Β_/dT* at λ = 1060 nm as well. It is also noted that the larger *ν_Β_* in 1060 nm SMFs may also assist in the efficient isolation of the Brillouin signal from the incident pump, which can serve as an advantage in optical signal processing. These results can be readily applied to existing distributed sensors using Brillouin scattering and provide a meaningful potential for application requiring a higher temperature sensitivity.

The strain dependence of Brillouin frequency shift, *dν_Β_/dε*, is another important parameter to be used in distributed fiber optic sensors. Usually, *dν_Β_/dε* has been measured using a high spatial precision Brillouin reflectrometry technique and the authors are investigating the strain dependence of 1060 nm SMFs, which will be published in a separate paper.

## 4. Conclusions

In conclusion, SBS characteristics of two passive single mode optical fibers operating at λ=1064 nm were experimentally investigated. The Brillouin frequency, *ν_Β_*, was measured to be 15.76 and 15.17 GHz in HI1060 and in HI1060FLEX fiber, respectively. The Brillouin linewidth, Δ*ν_Β_*, also showed a significant difference between the two fibers, 19.50 MHz in HI1060 and 26.03 MHz in HI1060FLEX. We observed the SBS threshold power *P_th_* of 50 and 24 mW for HI1060 and HI1060FlEX, respectively. The waveguide structure and the corresponding material composition in the two fibers were attributed to those differences in SBS characteristics. As a result of temperature-dependent SBS analyses, SBS frequency shift *dν_Β_/dT* was measured to be 1.83 MHz/°C for HI1060 and 1.72 MHz/°C for HI1060FLEX. The peak intensity of SBS also showed linear temperature dependence such that *dI_peak_/dT* was 0.11 and 0.16 dB/°C for HI1060 and HI1060FLEX, respectively. The temperature dependence in Brillouin frequency shift, *dν_Β_/dT*, in 1060 nm SMFs was found to be significantly larger than those in prior C-band SMFs by more than 40%, which can make available new highly sensitive distributed temperature sensing applications.

## Figures and Tables

**Table 1:** Comparison of temperature-dependent SBS characteristics.

Wavelength (nm)	Fiber Type	Fiber Length (m)	Temperature (°C)	*ν_Β_* (GHz)	*dν_Β_/dT* (MHz/°C)	Ref.
1064	HI1060 (Ge-doped core)	500	25 to 60	15.76	1.83	This work
1064	HI1060FLEX (Ge-doped core)	500	25 to 60	15.17	1.72	This work
1320	SMF (Ge-doped silica core)	200	−25 to 90	12.81	1.36	[43]
1550	SMF (Ge-doped silica core)	0.2	22 to 1000	10.85	1.35	[45]
1549	SMF (F-doped silica core/clad)	110	10 to 60	10.52	1.286	[36]
1550	Dispersion Shifted Fiber	110	20 to 820	10.53	1.25	[46]
1549	FutureGuide^TM^ (Ge-doped core)	10	10 to 60	10.87	1.12	[47]
1554	SMF	1650	39 to 77	10.9	1.1	[48]
1549	SMF (silica core)	50	10 to 60	11.17	1.075	[36]
1553	Dispersion Shifted Fiber	30	19 to 100	10.51	0.99	[41]
1550	SMF (Annealed Ge-doped core)	0.2	22 to 1000	10.85	0.42	[45]
